# Variations in Circulating Thyroid Hormone Profiles Across Age, Sex, and Pregnancy Outcomes in Killer Whales (*Orcinus orca*) Under Human Care

**DOI:** 10.3390/ani16060907

**Published:** 2026-03-13

**Authors:** Todd R. Robeck, Karen J. Steinman, Gisele A. Montano, Steve Paris, Janine L. Brown

**Affiliations:** 1 Species Preservation Laboratory, SeaWorld California, Inc., San Diego, CA 92109, USA; karen.steinman@unitedparks.com (K.J.S.); gisele.montano@unitedparks.com (G.A.M.); 2 United Parks and Resorts, Corporate Zoological Operations, Sea World Orlando, Orlando, FL 32821, USA; 3 Center for Species Survival, Smithsonian’s National Zoo & Conservation Biology Institute, 1500 Remount Road, Front Royal, VA 22630, USA; pariss@si.edu (S.P.); brownjan@si.edu (J.L.B.)

**Keywords:** thyroid function, thermoregulation, cetacean, pregnancy loss, reference ranges, Delphinidae

## Abstract

The study examined how season, age, sex, and pregnancy outcomes influenced serum total thyroxine (TT4) and triiodothyronine (TT3) levels in killer whales (*Orcinus orca*). Total T4 and TT3 concentrations were quantified in 1513 serum samples collected voluntarily over ~40 years from 14 males and 24 females (ages 1–54) under managed care. Data were analyzed using LMM to determine the effects of age, sex, season, and pregnancy status (normal vs. abnormal outcomes). Age, season, and pregnancy significantly influenced thyroid hormone concentrations, while sex did not. Juveniles exhibited higher concentrations consistent with increased thermoregulatory needs and growth demands. Seasonal analysis showed TT4 peaked in summer and declined in winter suggesting thermoregulatory adaptation. Pregnancies with abnormal outcomes (abortion, dystocia, stillbirth) were associated with atypical thyroid hormone profiles; specifically, dystocia was linked to consistently low TT3/TT4, while stillbirths correlated with elevated late-term TT3. Females experiencing abortion showed decreased TT3 and TT4 during the late gestation. These findings suggest that in pregnancies with adverse outcomes, metabolic imbalances or transient hyperthyroid-like states may negatively impact fetal health. Consequently, in killer whales, variation in thyroid hormone levels may reflect a complex interplay between environmental adaptation, reproductive status, and underlying evolutionary physiology.

## Simple Summary

This study establishes the first information for serum thyroid hormones in killer whales, which are vital for regulating metabolism and growth. Unlike some cetacean species, killer whales showed no hormonal differences between males and females. Key factors affecting these hormones in killer whales include: (1) age where younger animals have higher levels to support rapid growth, (2) season with hormone patterns characterized by increased consumption possibly linked to generating body heat, and (3) pregnancy with hormone concentrations being relatively increased during early gestation and then decreasing towards late pregnancy. In addition, the study found abnormal levels are linked to complications including miscarriages and stillbirths. Overall, the results show that killer whale thyroid secretion is influenced by environment, age and reproductive status.

## 1. Introduction

Research on thyroid hormones and thyroid gland function in cetaceans is limited, but some data exist for a few species. For killer whales, only one study has described fecal T3 metabolites, and none have characterized serum TH profiles [1]. Consequently, interpretations of killer whale fecal T3 hormone fluctuations have relied solely on comparative analysis against other species under the assumption that thyroid secretion, regulation and biologic activity are conserved across taxa. In general, thyroid hormones, thyroxine (T4) and triiodothyronine (T3) play pivotal roles in regulating normal physiology, either directly or indirectly through extensive interactions with other hormonal processes. They influence metabolism and energy availability, thermoregulation, growth and development, and homeostatic cellular functions across all major organ systems. These hormones, produced by the thyroid gland, are regulated by a complex feedback loop involving the hypothalamic–pituitary–thyroid (HPT) axis and peripheral deiodination, where circulating T4 is converted to the active form of T3 [2,3]. This conversion rate or rate of deiodination from T4 to T3 is reflected in the T3:T4 ratio. A high ratio is used clinically to detect hyperthyroidism or Graves’ disease, while a low ratio, without thyroid disease (euthyroid sick syndrome), is often the result of other physiologic stressors, e.g., nutritional deficiencies or chronic stress [4,5,6]. Understanding how this ratio fluctuates in a normal population of killer whales would be useful for evaluating overall wellness of animals both in the wild and managed care. Despite the central role of thyroid hormones in maintaining homeostasis, the factors driving thyroid dysregulation across mammalian taxa remain poorly understood, particularly for wildlife species. In marine mammals, environmental conditions demand extraordinary physiological adaptations, making thyroid function particularly important. Thyroid hormones help optimize energy utilization during deep dives, maintain thermal equilibrium in cold waters, and support reproductive processes [7,8].

Within marine mammals, studies have found that cetaceans have relatively large thyroid glands compared with domestic species [9]. This led to speculation that marine mammals produce higher amounts of T4 than other mammals [10]. Fair et al. (2011, Table 6) [11] collated published thyroid hormone concentrations across various taxa, including pinnipeds, and found that T4 concentrations were higher in beluga whales (*Delphinapterus leucas*) and bottlenose dolphins (*Tursiops truncatus*) than in other species, whereas T3 data were inconclusive.

In addition to relatively large thyroid glands, multiple thyroid abnormalities have been identified in both wild and aquarium-housed cetacean species. In wild bottlenose dolphins, reported pathologies include adenomas, hyperplastic nodules, cysts, interstitial fibrosis, amyloidosis, and thyroiditis [12]. A small number of pilot whales (*Globicephala* melas) from a mass stranding of 55 whales exhibited both macrofollicular goiter and granulomatous reactions indicative of inflammation [13]. Other thyroid abnormalities have been observed in situ in St. Lawrence River belugas, where exposure to industrial pollution was associated with an increased frequency of thyroid adenomas and hyperplasia [14]. In aquarium-housed bottlenose dolphins, pathological findings in periparturient neonates include diffuse hyperplastic goiter of unknown etiology [15]. Among adult female dolphins, those with live calves had significantly higher concentrations of total T4 (TT4), free T4 (FT4), and total T3 (TT3) than those experiencing perinatal loss [16]. Similarly, in wild Southern resident killer whales (*Orcinus orca*), fecal thyroid metabolites were lower in known pregnant females that were later observed without live calves or with calves that died shortly after birth than in pregnant females with live calves [1]. In that study, low thyroid hormone concentrations were correlated with reduced seasonal prey availability, suggesting that poor nutrition, rather than thyroid dysfunction alone, was the primary driver of reproductive failure [1]. Nutritional deficiency during gestation can independently impair fetal growth and development through intrauterine growth restriction in other species [17,18], with hypothyroidism being linked to poor fetal outcomes in humans and domestic animals [19,20]. But it remains unclear whether hypothyroidism acts as the primary cause of these outcomes or arises secondarily from the effects of poor nutrition.

In bottlenose dolphins, beluga and boto whales (*Inia geoffrensis*), age has been reported to be inversely correlated with thyroid production and to vary with season in wild and captive individuals [21,22,23]. In wild bottlenose dolphins, colder environmental or water temperatures correlated with increased thyroid production when compared between two distinct populations [11]. The same was also observed in wild beluga, where Arctic (Chukchi Sea) populations had higher TT3 and TT4 concentrations than subarctic, warmer-climate populations (Bristol Bay). For both species, higher TT4 may be a response to an increased need for heat production in the colder climates [11,21]. In contrast, both species exhibited lower TT4 concentrations during the winter months in aquarium settings [21,24]. However, in an additional group of bottlenose dolphins exposed to sub-tropical environmental conditions, no significant seasonal changes in TT4 were observed [24]. Findings across cetacean species regarding sex-related differences in thyroid hormone concentrations remain inconsistent, though some studies report differences during the juvenile period and again in adulthood [16,25]. Understanding how age, reproductive state, environmental temperature, and season influence thyroid function in killer whales is essential for establishing reliable physiological baselines. This information will contribute toward the interpretation of hormone dynamics for animals in managed care and may have broader applicability to wild populations.

The objective of this study was to determine if TT4 and TT3 concentrations and the TT3:TT4 in aquarium-housed killer whales was influenced by age, sex, pregnancy status and season. Additionally, we compared thyroid function between pregnant and non-pregnant females to determine if hormone patterns differed among females with poor reproductive outcomes—including abortion, stillbirth, dystocia, and calf mortality within 30 days. Finally, we identified reference ranges of thyroid hormone concentrations across the significant physiologic or environmental variables influencing their concentrations.

## 2. Materials and Methods

### 2.1. Animals and Management

Thirty eight killer whales housed at four SeaWorld parks (Orlando, FL, USA; Cleveland, OH, USA; San Diego, CA, USA; San Antonio, TX, USA) had blood collected during routine health assessments between 1977 and 2020 [26]. Animals were housed in mixed-sex and mixed-age groups (0 to ~55 y, Table 1) in habitats with a minimum of 19,000 m^3^ of salt water kept maintained between 12 °C and 16 °C. Animals were fed at approximately 2–3% of their body weight per day on a diet of whole fish [e.g., Atka mackerel (*Pleurogrammus azonus*), Columbia River smelt (*Thaleichthya pacifica*), Norwegian or Canadian Capelin (*Mallotus villosus*) Atlantic or Pacific herring (*Clupea* spp.), Pacific sardines (*Sardinops sagax*), and Pacific (*Oncorhynchus*, spp.) or Atlantic salmon (*Salmo salar*)]. All fish were graded for human consumption.

### 2.2. Sample Collection

Animals were conditioned to voluntarily permit blood sample collection (n = 1513) from ventral tail fluke veins on a routine bimonthly or monthly basis as part of SeaWorld’s veterinary health assessment program using a 19-gauge winged blood collection set into BD Vacutainer tubes (Becton Dickenson, Franklin Lakes, NJ, USA) containing activated thrombin. Samples were allowed to clot at room temperature for 20 min and then centrifuged at 1000× *g* for 15–30 min. Serum samples were aliquoted into multiple 1 mL cryotubes for long term storage at −80 °C until analysis to ensure hormonal stability over time [27,28]. No serum samples were used that had more than 1 previous freeze–thaw cycle [29].

### 2.3. Thyroid Hormone Analysis

Thyroid hormones were measured in serum using commercial solid phase radioimmunoassay (RIA) kits (MP Biomedicals, Los Angeles, CA, USA, TT3 catalog number TKT35; TT4 catalog number TKT45). All RIAs were conducted in accordance with the manufacturer’s instructions as previously described for use in boto [25], except for adding two additional low standards to the TT3 assay (standard range, 12.5 ng/dL to 800 ng/dL). Assays were validated for use in killer whales based on observed parallelism between serial dilutions of pooled serum samples, and the respective standard curves and >90% recovery of respective hormone standards from pooled male and female serum samples (Table S1). Assay sensitivities were 0.5 µg/dL for TT4 and 12.5 ng/dL for TT3. For all assays, any sample with a coefficient of variation between duplicates exceeding 10% was reanalyzed. Inter-assay variation for TT4 across four controls with antibody binding at approximately 65%, 50%, 35%, and 30% was 12.5%, 4.2%, 7.2%, and 5.9%, respectively (n = 10 assays). Inter-assay variation for TT3 for three controls with antibody binding at approximately 60%, 40%, and 30% was 8.8%, 3.5%, and 3.3%, respectively (n = 9 assays). Attempts to validate a thyroid-stimulating hormone (TSH) radioimmunoassay for use in killer whale serum were unsuccessful, and therefore TSH concentrations were not analyzed (Table S1). This result demonstrates the importance of assay validation prior to sample analysis for novel species and restricted our ability to interpret upstream regulation of thyroid hormone secretion.

### 2.4. Data Analyses

Animals were classified into age groups based on established maturity thresholds from prior studies [26,30]. Females younger than 8 years and males younger than 13 years were classified as juveniles. Samples from pregnant females were categorized based on known or estimated conception dates. Gestation or pregnancy stage (pstage) was divided into early (<179 days post-conception), mid (179 to 356 days), and late stage (>356 days) [31,32]. Pregnancy outcomes (pgresults) were classified as follows: normal (31 pregnancies in 13 animals, mean 11.2 ± 0.9 samples per pregnancy), production of a live calf that lived beyond 30 days; failure to thrive (three pregnancies in three animals, mean 10.7 ± 4.1 samples per pregnancy), live calves that died prior to 30 days; stillbirth (two pregnancies in two animals, mean 6.0 ± 2.8 samples per pregnancy), full term calves born dead or that died within 24 h; dystocia (three pregnancies in three animals, mean 14.0 ± 3.6 samples per pregnancy), stillborn calf within normal gestation length but clinically difficult labor; and aborted calf (three pregnancies in three animals, mean 11.0 ± 1.0 samples per pregnancy), dead calf born prior to the minimum gestation length (485 days) [32].

Data were analyzed using Stata^®^ (version 19; StataCorp LP, College Station, TX, USA) using a linear mixed-effect restricted maximum likelihood (REML) regression model to quantify the relationship between dependent (hormone data) and fixed-effect variables. Model development for the analysis of each hormone was initiated by first determining which random-effects structure (two- or three-level mixed-effects model) accounted for the greatest proportion of variance or provided the best model fit [33]. This was done by comparing a two-level REML using animal ID (repeated measures from each animal) with a three-level REML that controlled for animals nested within each housing location using a likelihood ratio (LR) test. Once the random-intercept structure of the models was determined, the effect of model fit with the addition of a random slope variable to each random intercept was evaluated using the LR test (Tables S2 and S4). Fixed-effects variables were evaluated to compare hormone concentrations across age groups, sex, and pregnancy, while controlling for season and air temperature. These fixed-effects categorical variables were: sex; season (winter [December through February], spring [March through May], summer [June through August], and fall [September through November]); and group (coded 0 to 4), which included juveniles (non-pregnant immature females < 8 yr, and males < 13 yr), adults (males and non-pregnant females), and pregnant animals classified as being in the early, mid, or late stage [32]. All pregnant animals in this initial group delivered live calves that survived at least 30 days postpartum. The continuous variable, monthly mean air temperature, was included. The full model included all fixed and random variables (as previously identified) and was then compared with reduced models in which each variable was iteratively removed. The model with the best fit was then determined based on the lowest Akaike information criterion (AIC) value [34] (Table S3).

For comparisons between animals with different pregnancy outcomes, the fixed categorical variables included: season; pstage (coded 0, 1, 2: representing early, mid or late stage, respectively); and pgresult (coded 0, 1, 2, 3: representing normal births, stillbirths, failure to thrive, dystocia, or abortion) and the interaction variable pstage*pgresult. The continuous variable age was included to control for potential age effects. As in the previous analysis, the optimal (final) model was determined by iteratively comparing the full model with reduced models in which variables were fixed, using AIC values (Table S5).

All final mixed models were checked for normality using quantile plots of the standard residuals. If quantile–quantile (qnorm) plots of standardized residuals exhibited non-normal distribution, the data were transformed as indicated by the Shapiro–Wilk test (Ladder command, Stata) for raw data until model residuals were normalized. The overall significance for each categorical variable was determined post hoc using a Wald test (testparm command, Stata). Pairwise comparisons of estimated marginal means were conducted using the margins command with Šidák correction or as paired contrasts against normal (in the case of normal versus abnormal pregnancy) without correction. Unless specified, data are presented as marginal means and 95% confidence intervals. For all analyses, *p* < 0.05 was considered significant.

Once significant groups (age, sex and pregnancy status) were identified by the linear mixed model (LMM) analysis, reference ranges for the central 90% range (5th, 25th, 50th, 75th, 95th percentiles) for each group were then calculated. To account for repeated measures within individuals, a nonparametric clustered bootstrap-based procedure was employed [35,36]. Using bootstrap resampling of individual IDs with replacement (1000 reps), the mean percentiles for each group were determined and the 95% bootstrapped confidence intervals (CIs) for each percentile were estimated [36].

## 3. Results

### 3.1. Overall Thyroid Hormone Concentrations

Across all ages, sexes, and pregnancies, mean TT3 was 0.090 ± 0.0010 µg/dL (0.090 to 0.093 µg/dL) and mean TT4 was 7.21 ± 0.057 µg/dL (7.10 to 7.32 µg/dL). On average, TT3 accounted for only 1.3% of the total immunoreactive thyroid hormone concentration, whereas TT4 contributed 98.7%.

### 3.2. Thyroid Hormone Concentrations Across Age, Sex, Season, and Gestation

The random-effects portion of the final models for TT3, TT4, and TT3:TT4 was significantly improved by including animal (ID) nested within animal location (park) as random intercepts in a three-level LMM. Additionally, stage as a random slope at level two and three intercepts significantly improved the fit of all three models (Table S2). Finally, we improved the models’ fit and variance estimation by defining residuals as independent across ID for TT3 (LR χ^2^ = 33, *p* < 0.0001), TT4 (LR χ^2^ = 51, *p* < 0.0001), and TT3:TT4 (LR χ^2^ = 32, *p* < 0.0001). The final fixed portion of the models used to analyze untransformed hormones across variables included mean monthly air temperature, sex, season of sample collection, and group (juvenile, non-pregnant adult, and normal pregnancy stages [early, mid, late]; Table S3). However, despite their inclusion, neither air temperature nor animal sex were significant in any of the analyses, while season was significant for both TT4 and TT3:TT4 with reduced marginal mean TT4 concentrations and increased TT3:TT4 in winter (7.3 µg/dL, 13.6 ng/µg, respectively) compared to other seasons; TT4 concentrations peaked during summer (8.10 µg/dL, Table 2).

In addition to season, the variable group was significant within all hormones analyzed (Table 3), and intra-group marginal means comparisons indicated that juvenile animals had significantly increased concentrations of TT3 (103.2 ng/dL) compared to all non-pregnant adults and all stages of pregnancy, while the late stage was reduced compared to all other groups (77.20 ng/dL, Table 3). For TT4, juvenile killer whales (8.72 µg/dL) had the highest concentrations compared to adults (6.81 µg/dL) and all stages of pregnancy; early pregnancy (7.65 µg/dL) was higher compared to adults (males and non-pregnant adult females) and late pregnancy (6.95 µg/dL, Table 3). For TT3:TT4, adult killer whales (14.25 ng/µg) had significantly higher concentrations compared to all other categories (Table 3). Further evaluation of this variable indicated that non-pregnant adult females were the primary driver for these significant differences, with females having an increased TT3:TT4 (15.1 ng/µg, *p* = 0.027) compared to adult males (13.0 ng/µg).

### 3.3. Thyroid Hormone Concentrations During Normal and Abnormal Pregnancies

The random-effects portion of the final models for TT3 and TT4 was significantly improved by including animal (ID) nested within animal location (park) as random intercepts in a three-level LMM (Table S4). Additionally, total T3 improved when park was included at the ID level as a random slope. The best model for TT3:TT4 was a two-level LMM with ID for random intercepts. No random slope variable improved the model fit. The final T4 model benefited from having residuals designated as independent by pstage (LR χ^2^ = 11, *p* = 0.0043), while the TT3:TT4 model was improved when residuals for pgresult were designated as independent (LR χ^2^ = 11, *p* = 0.0043).

Based on AIC analysis for each model, the final fixed portion of the models used to analyze untransformed TT3 and TT4 included female age, season, pstage (early, mid, and late), pgresult, and the interaction between pstage and pgresult (Table S5). The final TT3:TT4 model was similar but did not retain the interaction term. Age and season were significant controls for all models except TT3:TT4, where age was not significant (*p* = 0.08) but was retained as a control variable. For age, TT3 and TT4 decreased by 0.80 ± 0.20 ng/dL (*p* = 0.0001) and 0.028 ± 0.01 µg/dL (*p* = 0.014), respectively, for each additional year of age.

**Figure 1:**
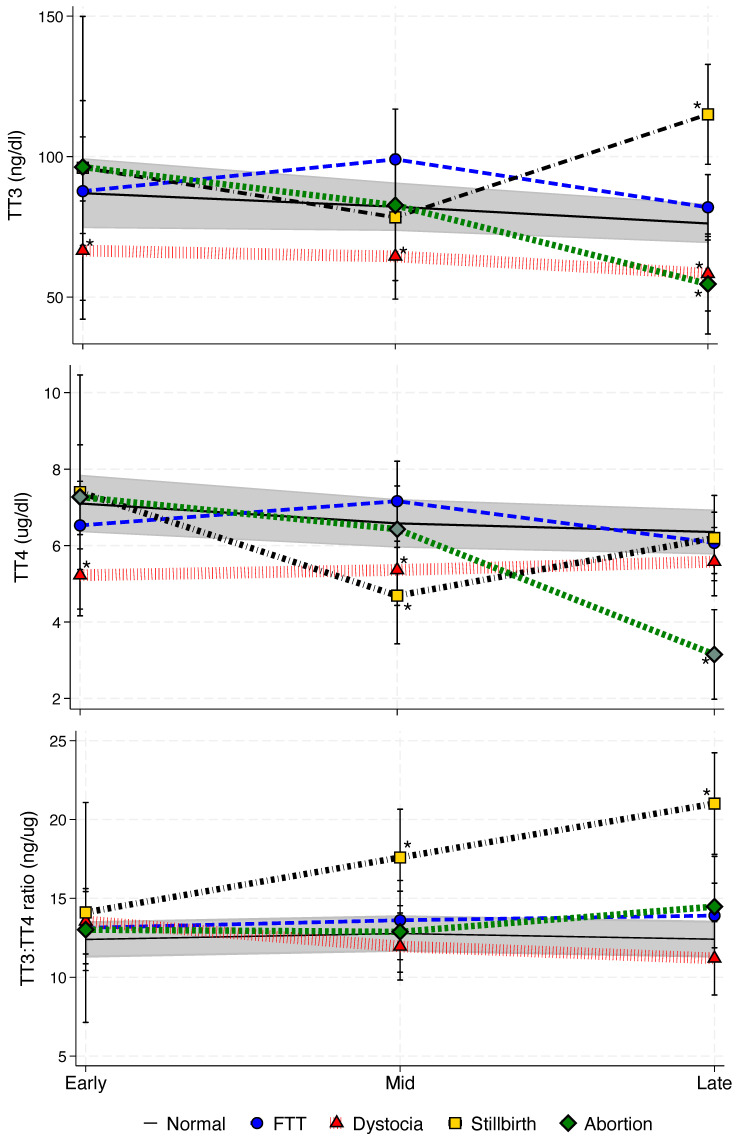
Serum concentrations of total triiodothyronine (TT3) and thyroxine (TT4) and the TT3:TT4 ratio during normal and abnormal pregnancies in 38 killer whales (*Orcinus orca*) sampled over 30 years at four SeaWorld parks. Normal pregnancies are represented by the grey shaded area representing the 95% confidence intervals for predict means. Abnormal pregnancy outcomes include failure to thrive (FTT; ●), dystocia (▲), stillbirth (◼︎) and abortion (⬥). Asterisks (*) indicate significant differences within each pstage (early, mid and late) against normal pregnancy state (*p* < 0.05).

For TT3, in addition to age and season (χ^2^ = 8.3, *p* = 0.04), pgresult was also significant (χ^2^ = 7.9, *p* = 0.049), but pstage was not. Within the pgresult variable, TT3 was lower in females with dystocia compared to normal births during early (χ^2^ = 7.3, *p* = 0.0068), mid (χ^2^ = 4.5, *p* = 0.0341), and late (χ^2^ = 7.2, *p* = 0.0073) gestation (Figure 1). During late pregnancy, females experiencing abortions (χ^2^ = 5.3, *p* = 0.0218) had lower TT3 concentrations, although for those with stillbirths (χ^2^ = 20.3, *p* < 0.0001) TT3 was increased (Figure 1).

For TT4, in addition to age and season (χ^2^ = 31.8, *p* < 0.0001), pstage (χ^2^ = 9.4, *p* = 0.0092), pgresult (χ^2^ = 18.9, *p* = 0.0003), and the pstage*pgresult interaction (χ^2^ = 38.2, *p* < 0.0001) were also significant. Compared with normal births, dystocia was associated with lower concentrations during early gestation (χ^2^ = 17.0, *p* < 0.0001). During mid-gestation, TT4 concentrations were reduced in both dystocia (χ^2^ = 9.4, *p* = 0.0022) and stillbirth (χ^2^ = 10.1, *p* = 0.0015) cases (Figure 1). In late gestation, only abortion (χ^2^ = 30.3, *p* < 0.0001) was associated with lower TT4 concentrations (Figure 1).

Overall, the TT3:TT4 varied only by season (χ^2^ = 41.2, *p* < 0.0001). However, among pgresults, the ratio was higher for stillbirths than for normal births at both mid (χ^2^ = 9.8, *p* = 0.0018) and late (χ^2^ = 28.9, *p* < 0.0001) stage (Figure 1).

### 3.4. Thyroid Hormone References Ranges

Percentiles (5th, 25th, 50th, 75th, and 95th percentiles) and the associated 90% bootstrap (1000 reps) confidence intervals (CI) for thyroid hormones by age class (juvenile vs adult) and pregnancy status are provided in Table 4. As noted above, no significant differences were detected between sexes, so data were pooled to generate reference ranges. In general, juveniles had higher TT3 and TT4 concentrations than adults and a lower TT3:TT4. Both TT3 and TT4 decreased during pregnancy, although the ratio remained unchanged. The highest TT3:TT4 was found in adult killer whales (Table 4).

## 4. Discussion

These results present the first comprehensive description of serum TT3 and TT4 concentrations in killer whales across age, season, sex, and pregnancy states. Thyroid hormone reference ranges for killer whales also were established to provide baseline concentrations across life stage and to support health assessments, comparative physiological studies, and the identification of physiological and environmental factors influencing thyroid function. Using comparable methodologies, serum TT4 concentrations in these killer whales were lower than those reported in bottlenose dolphins [16], but higher than those in botos [25]. Unlike observations in the other two species, no sex differences were detected. As with TT4, TT3 concentrations were nearly twice those observed in boto across all age groups and pregnancy stages but were comparable to bottlenose dolphin concentrations in juvenile and adult animals.

Within these cetacean species, body mass is a major distinguishing factor. On average, an average adult killer whale weighs approximately 10 times the body mass of bottlenose dolphins, which in turn weigh about twice that of boto. Across various taxa, smaller body mass is associated with a higher mass-specific metabolic rate, known as metabolic allometry [8,37,38]. Within this framework, smaller mammals exhibit a higher metabolic rate per gram of body weight. Increased thermoregulatory demands are hypothesized to be primary drivers because smaller animals have a larger surface-area-to-body-mass ratio, which increases environmental heat loss. Metabolic rates and their role in thermoregulation are believed to be primarily regulated by the hypothalamo–pituitary–thyroid axis, which controls the release and conversion of thyroid hormones, specifically T4 to T3, through a process called deiodination [2]. Smaller cetacean species are expected to have higher relative metabolic rates [39] and, consequently, elevated thyroid hormone levels. When comparing killer whales and bottlenose dolphins, an allometric relationship was observed, with bottlenose dolphins having higher thyroid concentrations across age and sex classes. However, unexpectedly, and based solely on metabolic allometry, the much smaller boto had thyroid concentrations approximately half those of bottlenose dolphins and killer whales (Table 5). These differences may be due, in part, to the thyroid’s role in adaptive thermogenesis [2] which would manifest as variations in baseline TH secretion in response to normal temperature fluctuations for species whose range includes different habitats. For instance, in other species, increased environmental temperatures are associated with reduced thyroid hormone production [40,41]. Evidence supporting environmental adaptations as the primary source of these differences exists in bottlenose dolphins, where thyroid concentrations are influenced by location and inversely related to the mean ambient water temperature of their primary habitat [11,24].

Iodine availability in food sources may also influence thyroid hormone production. Since iodine is abundant in the marine species and thus a diet consisting of marine fish, as is the case with our killer whales, should provide an adequate supply of iodine. Conversely, the boto diet consists of freshwater fish, which have lower iodine concentrations [42,43] that may vary by season [44,45]. Therefore, the availability of dietary iodine could potentially limit the production of thyroid hormones. Low environmental iodine levels are well documented to cause hypothyroidism and goiter in both humans and animals, leading to numerous detrimental developmental and functional effects [46,47,48]. However, no goiter-related issues have been observed in the boto population, and based solely on our lower comparative thyroid concentration observations [25], the species would not be considered hypothyroid. It is likely that some evolutionary adaptations have occurred, enabling freshwater dolphins (at least within the boto species) to tolerate the low iodine content in their food sources. Rodents that have evolved in chronically low-iodine environments do not exhibit lower TT4 concentrations, suggesting they have developed mechanisms to maintain normal T4 concentrations through enhanced iodine absorption and heightened sensitivity to T3 [49]. Thus, the comparatively lower thyroid hormone production observed in boto remains unexplained. In killer whales, the predominant circulating hormone was TT4 at 98.7%, with TT3 accounting for only 1.3%. This distribution is identical to that observed for the boto [25] and similar (98 to 99.2% TT4) to bottlenose dolphins [11,16].

### 4.1. Effect of Sex and Age

There were no significant differences in thyroid hormone concentrations in killer whales between sexes at any age. These results are similar to those reported for wild bottlenose dolphins [11], but different from aquarium-housed bottlenose dolphins, where significant sex differences in both juveniles and adults have been observed [16]. Additionally, sex-related differences in both TT3 and TT4 exist across all age groups in wild boto [25] and beluga [21]. These differences may be partly driven by sex steroid hormones, androgens or estrogens, during sexual maturation. However, since we did not directly compare changes in reproductive steroid hormones against TH concentrations in this study, we cannot say if changes in circulating sex hormone concentrations in killer whales have an effect on TH.

Sexual dimorphism occurs in cetaceans, with adult male killer whales, belugas, and botos being up to twice the size of females. Under a theoretical thermogenesis model, smaller females with a higher surface-area-to-mass ratio, particularly in aquatic environments, should require higher thyroid hormone concentrations to increase metabolic rate and maintain body heat. Despite these size differences, no significant sex differences were detected in killer whales. As previously mentioned, in beluga whales, males have higher TT4 levels, but these differences are limited to the breeding season [21]. If body mass is the primary factor driving thermogenic thyroid production, seasonal changes that align primarily with seasonal reproduction rather than environmental changes should not be detected. Clearly, further research is needed to understand the interplay among body mass, androgen production, seasonality, and thyroid hormones across cetacean taxa.

In killer whales and in previously studied cetaceans, juveniles exhibit higher thyroid hormone concentrations. Similar to that reported in beluga [21], we observed juvenile killer whales had both elevated TT3 and TT4 compared to adults, but for boto and bottlenose dolphins, only juvenile males had increased concentrations [16,21,25]. However, the TT3:TT4 ratio was higher in killer whale adults compared to juveniles. In a population of endangered, wild killer whales, fecal thyroid metabolites, presumptively representing primarily circulating T3 were elevated in juveniles compared to adults [1]. This age-influenced increase in thyroid hormone production is commonly observed across mammalian taxa, including humans, and most likely is a consequence of both the need for increased metabolic demands for thermogenesis within smaller juveniles and the energetic demands of growth [50,51,52].

### 4.2. Effect of Season

Similar to beluga whales [21] and bottlenose dolphins [24], seasonal variations in thyroid hormones were observed in killer whales. Total T4 concentrations peaked during the summer and were lowest in winter, while concentrations of T3 remained relatively consistent across seasons; the TT3/TT4 ratio increased during the winter months. This suggests that peripheral tissues may have increased the conversion of T4 to T3 in response to the heightened metabolic demands of colder environments. The relative stability of circulating TT3 despite these demands likely represents an accelerated T3 turnover rate, where peripheral production is offset by rapid tissue utilization. Typically, T3 concentrations, which include total, free, and reverse T3, are tightly maintained via an increased or decreased production of T3 and T4 within the thyroid gland or peripheral deiodination of T4 at the tissue level in response to metabolic and thermoregulatory changes in demand [52]. The relatively high concentration of TT4 in killer whales and other cetaceans as compared to many terrestrial mammals [11,52] may represent an important reserve for seasonal or acute changes in ambient water, and air temperatures and food availability. In wild killer whales, fecal TT3 metabolite concentrations decrease during the summer months and were inversely related to changes in annual prey availability [53]. Because we did not observe significant seasonal changes in TT3 (only TT4) in the presence of a steady food supply, our results support the assumption that the changes observed in wild killer whale fecal TT3 are primarily driven by prey availability rather than seasonal changes.

### 4.3. Effect of Pregnancy Status

There were significant increases in TT4 concentrations during early gestation compared to non-pregnant females, but TT3 concentrations remained unchanged. This contrasts with boto, where TT3 concentrations were lower in early gestation compared to non-pregnant females. In killer whales, after an initial post-conception increase, both TT4 and TT3 decreased during mid-gestation, with a significant reduction observed in the late pregnancy. Similarly, in boto and bottlenose dolphins, TT3 also decreased throughout each stage of pregnancy [11,16,25]. Similar decreases during pregnancy have also been observed in aquarium-housed bottlenose dolphins, in which significant increases in both TT3 and TT4 during early pregnancy relative to non-pregnant adults occur [16]. This consistent pattern of decreasing thyroid hormone levels from early to late pregnancy, observed in three cetacean species, has also been reported in goats [54] and cattle [55]. However, unlike in cetaceans, T4 concentrations in cattle increase throughout gestation. Further differences are observed in humans and non-human primates in which peak thyroid concentrations occur during the mid stage of pregnancy [56].

Similar to wild killer whales and bottlenose dolphins [1,16], differences in thyroid hormone concentrations were observed in females that experienced poor reproductive outcomes. For wild killer whales, because recovering a dead fetus or calf is rare and conception date determination is near impossible, abortions, stillbirths, and calves that experience death prior to 30 days (FTT) are categorized as reproductive loss [1]. However, these varied events often have different etiologies. Mid- to late-term abortions can be caused by infections or inflammation within the maternal and/or fetal environment. Additionally, these fetal losses may be attributed to maternal nutritional deficiencies leading to abnormal chorionic villus or poor corpora lutea development which can lead to insufficient nutritional support for normal fetal development [20,32,57,58,59]. By contrast, stillbirths are considered fully developed calves that typically die either shortly before or during birth. They may also be caused by an acute maternal or fetal inflammatory event, but are more commonly due to some form of oxygen deprivation during labor, such as dystocia or umbilical cord entrapment [60,61]. Wasser et al. (2017) [1] determined that changes in fecal TT3 metabolite concentrations correlated with prey availability in Southern resident killer whales. In addition, they determined that a lack of prey adversely impacted fetal health leading to an increase in reproductive failures. Consistent with our findings, West et al. (2014) [16] found low TT3 and TT4 for non-successful births but did not attempt to further classify the type of pregnancy loss or reproductive failure. We observed that significant fluctuations in TT3 concentrations in killer whales can also be associated with reproductive loss. For example, lower TT3 and TT4 concentrations throughout gestation were observed in three apparently healthy females of normal reproductive age that experienced dystocia. Histopathology reports from 10 deceased neonatal bottlenose dolphin calves revealed diffuse hyperplastic goiter, suggesting low thyroid concentrations among these calves; dystocia was speculated to be the cause of at least some of these losses [15]. Low TT3 has been associated with dystocia, primarily due to decreasing or impaired uterine contractility. The mechanisms involved include reduced uterine sensitivity to oxytocin, prostaglandin production, and loss of myometrial functionality [62,63,64]. Thus, similar mechanisms might have been responsible for the observed dystocia during labor in our three killer whales.

All three abortions showed evidence of placentitis via ascending infections that led to fetal death. Systemic illness was observed only during one of these incidents. Ascending infections are common causes of abortions in multiple domestic species [65,66]. However, because thyroid hormones were only decreased during late pregnancy, that probably was not the primary cause of fetal loss. Evidence has long existed that inflammation and cytokines released during inflammation can negatively impact thyroid production [67,68], while recent evidence in pigs provides a direct link between highly infected fetuses (viral infection) and significant reductions in T3 and T4 concentrations in both the fetus and the sow [69]. However, despite such evidence in other species, the relationship between thyroid hormones and killer whale reproductive failure remains speculative.

Among females with stillbirths, TT3 concentrations increased significantly during late gestation, accompanied by an increase in TT3:TT4. To our knowledge, hyperthyroidism has not been reported in cetaceans. However, thyroid adenomas, hyperplasia, or other tumors believed to be linked to environmental contaminants have been identified during post-mortem exams in wild beluga within the St. Lawrence River population. These lesions can cause hyperthyroid conditions in dogs and horses [14,70,71]. In our study, except for late gestation in stillbirth pregnancies, we found no evidence of hyperthyroidism in the killer whales. Generally, hyperthyroidism during pregnancy has been associated with low birth weight and fetal developmental abnormalities in humans, dogs, and cats [72,73]. Our findings suggest an increase in the conversion of T4 to T3 or a significant decrease in T4 production begins mid-pregnancy during stillbirth outcomes. By the end of gestation, the mean T4 concentrations approached the lower limit of the 95% confidence interval for normal pregnancies. This suggests that some metabolic imbalance was occurring during these abnormal pregnancies, leading to an overcompensation of T4 conversion to T3 during the final stage of gestation, and coincides with the period of maximum fetal growth. In humans, a classic presentation of increased T3 levels, characterized by elevated T3:T4 and decreased TSH (which we were unable to accurately measure in this study) is associated with a syndrome known as a thyroid storm [74]. Thyroid storms can be triggered during pregnancy by abnormally high chorionic gonadotropin concentrations (hCG), Graves’ disease, acute systemic infection, or parturition. If left untreated, thyroid storms can progress to a critical medical crisis [74,75]. Transient abnormal increases in hCG can also cause gestational transient thyrotoxicosis that, when prolonged, can lead to a thyroid storm [76]. Graves’ disease, an autoimmune disorder, can cause elevated thyroid concentrations during pregnancy (or non-pregnancy). However, it has only been identified in humans [77], and there were no clinical presentations that suggested a similar syndrome occurred during these pregnancies. Cats are well-known to have hyperthyroidism, which is not limited to pregnancy and becomes more common as they age. However, beyond tumors and possible dietary factors, no definitive causes have been identified [73,78,79]. Because these killer whales were clinically normal and delphinids do not appear to produce significant amounts of a chorionic gonadotropin [80], we cannot explain this pattern of hyperthyroidism during late pregnancy. However, its significant association with stillborn calves warrants further investigation.

Unfortunately, we were unable to measure thyroid-stimulating hormone (TSH). Detecting TSH may have helped us understand some of the thyroid changes we observed in this study. TSH is considered the gold standard for diagnosing human thyroid function because it remains relatively stable under various external stressors, such as nutritional, environmental, or psychological factors and accurately reflect the body’s true biological needs [81]. Additionally, because of its sensitivity, TSH can be used as an early indicator of abnormal thyroid function [82]. Future efforts to identify or develop a cetacean-specific TSH assay may provide further insights into our theories.

## 5. Conclusions

This study provides the first description of TT3 and TT4 in killer whale serum, establishing relevant reference ranges across different physiologic states and seasons for this killer whale population that can be compared to changes observed in other cetacean species with thyroid hormone data. Median TT4 concentrations in killer whales were lower than in bottlenose dolphins but higher than in botos, whereas TT3 concentrations were approximately double those in botos and similar to those in bottlenose dolphins. Unlike botos and bottlenose dolphins, no sex differences in thyroid concentrations were detected in killer whales. Our findings do not fully align with predictions based on metabolic allometry, suggesting that additional ecological or physiological factors may influence thyroid hormone concentrations in cetaceans. For example, the smaller boto was expected to have higher thyroid concentrations, but instead exhibits lower concentrations, possibly reflecting adaptations to warm freshwater environments, reduced thermogenic demand, and lower dietary iodine.

Age, season, and pregnancy significantly influenced thyroid hormone concentrations. Juvenile killer whales exhibited higher TT3 and TT4 compared to adults, aligning with their increased metabolic and growth demands. Seasonal analysis revealed that TT4 peaked in summer and declined in winter, while TT3:TT4 rose in winter. This suggests increased TT4 conversion, possibly to support thermogenic needs. During pregnancy, TT4 was higher in early gestation, then gradually decreased and remained stable from mid to late gestation. Conversely, TT3 decreased from early to mid and from mid to late gestation. Abnormal reproductive outcomes were associated with atypical thyroid profiles. Dystocia and abortions were linked with low TT3, while stillbirths were associated with elevated total TT3 and TT3:TT4 late in pregnancy. These findings suggest that there may be metabolic or inflammatory imbalances or temporary hyperthyroid-like conditions that negatively impacted pregnancy outcomes. Overall, the variations in killer whale thyroid patterns reflect a complex interplay of factors, including body mass, environmental adaptations, reproductive status, and evolutionary physiology.

## Figures and Tables

**Table 1:** Population demographics of the SeaWorld killer whales (n = 38) that contributed samples for this study.

	Female (n = 24)		Male (n = 14)		Total	
	Mean ± SD y (Range)	No of Animals ^1^ (Total Samples)	Mean ± SD y (Range)	No of Animals ^1^ (Total Samples)	Mean ± SD y (Range)	No of Animals ^1^ (Total Samples)
Group						
Juvenile	3.8 ± 1.3 (1.0 to 5.9)	13 (108)	4.8 ± 1.8 (0.9 to 8.0)	11 (234)	4.5 ± 1.8 (0.9 to 8.0)	24 (342)
Pubescent	6.6 ± 0.9 (5.1 to 8.0)	12 (33)	10.4 ± 1.4 (8.0 to 12.9)	11 (188)	9.8 ± 1.9 (5.1 to 12.9)	23 (221)
Adult	21.2 ± 11.9 (8.1 to 54.3)	21 (188)	21.0 ± 6.6 (13.0 to 38.8)	9 (312)	21.1 ± 8.9 (8.1 to 54.3)	30 (500)
Pregnant	17.2 ± 7.4 (5.9 to 37.1)	17 (450) ^2^			17.2 ± 7.4 (5.9 to 37.1)	17 (450)
Total	15.9 ± 9.9 (1.0 to 54.3)	63 (779)	13.1 ± 8.4 (0.9 to 38.8)	31 (734)	14.5 ± 9.3 (0.9 to 54.3)	94 (1513)

^1^ Total number of animals that contributed to each group. Since sampling was longitudinal, animals would be included in more than one age group. ^2^ Seventeen females across 42 pregnancies.

**Table 2:** Seasonal concentrations (mean ± SEM; 95% confidence intervals) of serum total triiodothyronine (TT3), total thyroxine (TT4), and the TT3:TT4 ratio (×1000) for 38 killer whales (*Orcinus orca*) sampled over 30 years at four SeaWorld parks, adjusted for sex, age, and pregnancy status.

Group	TT3 (ng/dL)	TT4 (µg/dL)	TT3:TT4 (ng/µg)
Variable stats (Chi Sq, *p* value)	1.8, 0.63	51, <0.0001	32, <0.0001
Winter (A)	94.3 ± 6.10 (82.4 to 106.2)	7.27 ± 0.29 (6.7 to 7.83)	**13.63 ± 0.46** (12.73 to 14.54)
Spring (B)	94.1 ± 6.0 (82.3 to 106)	7.65 ± 0.29 (7.09 to 8.21)	12.9 ± 0.46 (12 to 13.8)
Summer (C)	95.7 ± 6.0 (83.9 to 107.5)	**8.07 ± 0.29** (7.51 to 8.63)	12.31 ± 0.46 (11.42 to 13.21)
Fall (D)	93.4 ± 6.0 (81.6 to 105.2)	7.7 ± 0.29 (7.13 to 8.25)	12.7 ± 0.46 (11.81 ± 13.6)
Šidák groups	NS	A < B, D < C	B, C, D < A

NS: no significant differences. Šidák groups: Šidák correction factor used for comparisons of marginal means. Only stages that are significantly different (*p* < 0.05) from other groups are in bold font.

**Table 3:** Serum triiodothyronine (TT3), total thyroxine (TT4), and the TT3:TT4 ratio ×1000) marginal mean ± sem (95% confidence interval) concentrations from 53 killer whales (*Orcinus orca*) over 30 years at 4 SeaWorld Parks.

	Hormone		
Groups	TT3 (ng/dL)	TT4 (µg/dL)	TT3:TT4 (ng/µg)
Variable stats (Chi Sq, *p* value)	43, <0.0001	75, <0.0001	41, <0.0001
Juvenile (A) (n = 563)	**103.2 ± 6.2** (91 to 115.4)	**8.72 ± 0.28** (8.2 to 9.3)	12.28 ± 0.43 (11.44 to 13.11)
Nonpregnant adult (B) (n = 501)	91.1 ± 6.5 (78.2 to 103.9)	6.81 ± 0.36 (6.11 to 7.5)	**14.25 ± 0.61** (13.05 to 15.44)
Pregnant			
Early (C) (n = 152)	89.9 ± 6.8 (76.4 to 103.3)	7.65 ± 0.36 (6.94 to 8.36)	11.84 ± 0.68 (10.50 to 13.18)
Mid (D) (n = 155)	86 ± 6.8 (72.7 to 99.2)	7.27 ± 0.34 (6.61 to 7.93)	11.96 ± 0.6 (10.78 ± 13.14)
Late (E) (n = 142)	77.2 ± 6.8 (63.9 to 90.4)	6.95 ± 0.34 (6.29 to 7.61)	11.51 ± 0.59 (10.35 to 12.67)
Sidak Groups	E < B, D < A E < B	B, C, D, E < A B, E < C	A, C, D, E < B

Juvenile (female < 8, Male < 13), Early pregnancy (Day 2 through 178 post-conception [pc]), Mid pregnancy (Day 179 to day 356 pc) and Late pregnancy (day 357 until parturition, [32]). Sidak correction factor used for comparisons of marginal means. Only stages that were significantly different (*p* < 0.05) from all other groups are in bold font.

**Table 4:** Serum thyroid hormone (total T3 ng/dL, total T4 µg/dL, T3:T4 ratio ng/µg) references ranges (5th, 25th, 50th, 75th, and 95th percentiles) and the associated 90% bootstrap (1000 reps) confidence intervals (CI) for killer whale (*Orcinus orca*) serum samples (n = 1517) collected from 1977 to 2021 and during 28 normal pregnancies.

TH	Percentile	Juvenile (n = 562)	Non-Pregnant Adult (n = 502)	Early (n = 152)	Mid (n = 155)	Late (n = 142)
TT3	5	45.3 ± 10.7 (30.2 to 68.9)	42.5 ± 6.1 (37.8 to 61.5)	42.6 ± 2.3 (39.1 to 49.2)	44.7 ± 3.0 (39.5 to 53.2)	46.6 ± 2.3 (43.2 to 52.4)
	25	79.6 ± 8.0 (62.5 to 92.6)	67.6 ± 8.0 (53.4 to 82.9)	56.5 ± 6.3 (49.2 to 72.1)	61.1 ± 3.5 (55.5 to 67.8)	58.3 ± 2.4 (55.2 to 64.6)
	50	103.0 ± 6.3 (91.6 to 116.0)	88.8 ± 8.2 (71.6 to 102.2)	80.1 ± 7.1 (66.1 to 128.0)	71.9 ± 2.9 (68.9 to 94.5)	68.9 ± 4.4 (63.2 to 78.1)
	75	126.8 ± 4.5 (116.0 to 134.0)	112.3 ± 5.5 (97.8 to 121.8)	97.6 ± 10.8 (88.1 to 128.0)	97.2 ± 5.7 (87.4 to 108.9)	85.0 ± 6.4 (74.8 to 99.5)
	95	155.7 ± 5.2 (145.6 to 165.8)	141.6 ± 5.3 (130.1 to 151.4)	137.0 ± 8.7 (121.5 to 154.0)	128.3 ± 11.7 (110.6 to 165.9)	118.8 ± 12.6 (98.8 to 143.7)
TT4	5	5.7 ± 0.3 (5.4 to 6.4)	4.1 ± 0.3 (3.5 to 4.7)	3.5 ± 0.5 (3.0 to 4.9)	3.9 ± 0.4 (3.0 to 4.5)	4.0 ± 0.2 (3.5 to 4.2)
	25	7.3 ± 0.3 (6.6 to 7.9)	5.4 ± 0.3 (4.7 to 5.9)	5.4 ± 0.4 (4.7 to 6.1)	5.0 ± 0.3 (4.5 to 5.5)	4.8 ± 0.2 (4.4 to 5.3)
	50	8.6 ± 0.3 (8.0 ± 9.1)	6.4 ± 0.3 (5.8 to 6.9)	6.4 ± 0.4 (5.8 to 7.3)	5.8 ± 0.3 (5.3 to 6.5)	5.6 ± 0.2 (5.2 to 6.1)
	75	9.9 ± 0.4 (9.2 to 10.7)	7.3 ± 0.3 (6.9 to 8.1)	7.7 ± 0.6 (7.0 to 9.0)	6.8 ± 0.5 (6.2 to 7.8)	6.3 ± 0.4 (6.0 to 7.4)
	95	12.8 ± 0.6 (11.6 to 13.9)	9.0 ± 0.3 (8.4 to 9.5)	9.8 ± 0.4 (8.7to 10.3)	9.0 ± 0.6 (8.7 to 10.0)	8.3 ± 0.7 (7.2 to 10.0)
TT3:TT4	5	6.2 ± 1.0 (4.6 to 8.5)	6.3 ± 1.6 (4.9 to 9.9)	7.2 ± 0.7 (5.9 to 8.7)	8.5 ± 0.4 (7.4 to 9.1)	8.2 ± 0.5 (7.4 to 9.5)
	25	9.6 ± 0.7 (8.1 to 10.7)	11.3 ± 1.5 (7.6 to 13.5)	10.2 ± 0.4 (9.3 to 11.5)	11.1 ± 0.5 (9.7 to 11.6)	11.1 ± 0.6 (9.9 to 12.1)
	50	11.6 ± 0.5 (10.7 ± 12.7)	14.4 ± 1.1 (11.8 to 16.0)	12.6 ± 0.4 (11.7 to 13.2)	13.0 ± 1.0 (11.8 to 14.4)	12.9 ± 0.5 (11.6 to 13.8)
	75	14.1 ± 0.5 (13.3 to 15.2)	17.4 ± 1.0 (15.2 to 19.1)	14.4 ± 0.4 (13.4 to 15.2)	16.0 ± 1.0 (14.1 to 18.1)	14.6 ± 1.1 (13.2 to 18.0)
	95	17.6 ± 0.5 (16.5 to 18.6)	21.9 ± 1.4 (19.5 to 25.7)	20.2 ± 2.3 (16.2 to 23.3)	21.3 ± 1.6 (18.1 to 23.4)	21.4 ± 1.1 (16.9 to 22.2)

Juvenile (female < 8, Male < 13), early pregnancy (Day 2 through 178 post-conception [pc]), mid (Day 179 to day 356 pc) and late (day 357 until parturition [32]). Normal pregnancies were animals with live calves living beyond 30 days post-partum.

**Table 5:** Comparisons of thyroid hormone concentrations between Amazon River dolphin (Boto, *Inia geoffrensis*), common bottlenose dolphin (*Tursiops truncatus*), and killer whales (*Orcinus orca*) across sex and age groups, and pregnancy states.

	Common Name	Total T3 (ng/dL) 50th % (25–75%)	Total T4 (µg/dL) 50th % (25–75%)
**Age class**			
Juvenile			
Male	Boto ^1^	57 (43–90)	4.2 (3.5–5.1)
	Killer whale ^2^	104 (84–127)	8.5 (7.3–9.7)
	Bottlenose dolphin ^3^	90 (80–110)	13.9 (11.6–16.7)
Female	Boto	46 (32–52)	4.4 (3.9–4.7)
	Killer whale	112 (87–135)	8.7 (7.4–11.2)
	Bottlenose dolphin	117 (100–200)	18.4 (14.7–19.9)
Adult			
Male	Boto	59 (43–59)	3.3 (2.9–4)
	Killer whale	87 (61–111)	6.6 (5.8–7.4)
	Bottlenose dolphin	80 (60–100)	9.5 (7.7–12.8)
Female	Boto	49 (41–65)	4 (3–4.9)
	Killer whale	93 (72–113)	5.8 (4.7–7.1)
	Bottlenose dolphin	90 (70–120)	12.1 (9.6–14.3)
Pregnancy stage			
Early	Boto	45 (37–50)	4.3 (3.4–4.7)
	Killer whale	80 (57–98)	6.4 (5.4–7.8)
	Bottlenose dolphin	120 (90–150)	14.3 (12.8–16.6)
Mid	Boto	37 (32–43)	3.8 (3.7–4.5)
	Killer whale	72 (61–97)	5.8 (5–6.8)
	Bottlenose dolphin	100 (90–150)	13.6 (12.0–15.4)
Late	Boto	37 (34–41)	3.8 (3.2–4.6)
	Killer whale	69 (58–85)	5.6 (4.8–6.3)
	Bottlenose dolphin	90 (80–120)	11.4 (9.8–13.5)

^1^ Robeck et al., 2019 [25], ^2^ Present study; ^3^ West et al., 2014 [16].

## Data Availability

The data presented in this study are available on reasonable request from the corresponding author.

## Supplementary Materials

The following supporting information can be downloaded at: https://www.mdpi.com/article/10.3390/ani16060907/s1, Table S1. Assay validation statistics for thyroid hormone assays: Total triiodothyronine (T3); thyroxine (T4); and thyroid stimulating hormone (TSH). Assay validations included parallelism and accuracy check for two pools of killer whale serum samples, one female and one male. TSH parallelism did not pass validation threshold, so the accuracy/recovery was not performed. Table S2. Evaluation of the random effects portion of the restricted maximum likelihood linear mixed model (LMM) analysis of the killer whale thyroid hormones total triiodothyronine (tT3), total thyroxine (tT4) and their ratio (tT3:tT4). The random effects was first evaluated to determine if model was improved using a 2 (animal id) or 3 (animal nested within location) level mixed effect model (REML). All fixed variables were included during the selection of the random portion of the models. Models containing variables for random slopes were then compared to models without only random intercepts. Significance of full and reduced models were assessed using LR test. Final models are in bold. Table S3. Fixed variable selection for the 3-level restricted maximum likelihood linear mixed model (REML) analysis of the killer whale thyroid hormones total triiodothyronine (tT3), total thyroxine (tT4) and their ratio (tT3:tT4) with animal location (park) and animals as the random intercept variable, group as random slopes and residuals independent by group. Final fixed portion of the model was selection based on the model having the lowest AIC, BIC and CAIC. Fixed portion of model variables listed prior to parallel lines, random portions listed after. Table S4. Evaluation of the random effects portion of the restricted maximum likelihood linear mixed model (LMM) analysis of the killer whale thyroid hormones, total triiodothyronine (tT3), total thyroxine (tT4) and their ratio (tT3:tT4) within normal and abnormal pregnancies. The random effects was first evaluated to determine if model was improved using a 2 (animal id) or 3 (animal nested within location) level mixed effect model (REML). All fixed variables were included during the selection of the random portion of the models. Models containing variables for random slopes were then compared to models without only random intercepts. Significance of full and reduced models were assessed using LR test. Final models are in bold. Table S5. Fixed variable selection for the 3-level restricted maximum likelihood linear mixed model (REML) analysis of the killer whale thyroid hormones total triiodothyronine (tT3), total thyroxine (tT4) and their ratio (tT3:tT4) within pregnancy. Animal location (park) and animals as the random intercept variable and stage as random slopes. Final fixed portion of the model was selection based on the model having the lowest AIC, BIC and CAIC. Fixed portion of model variables listed prior to parallel lines, random portions listed after.

## References

[1] Wasser S.K., Lundin J.I., Ayres K., Seely E., Giles D., Balcomb K., Hempelmann J., Parsons K., Booth R. (2017). Population growth is limited by nutritional impacts on pregnancy success in endangered Southern Resident killer whales (*Orcinus orca*). PLoS ONE.

[2] Mullur R., Liu Y.-Y., Brent G.A. (2014). Thyroid hormone regulation of metabolism. Physiol. Rev..

[3] Ortiga-Carvalho T.M., Chiamolera M.I., Pazos-Moura C.C., Wondisford F.E. (2011). Hypothalamus-pituitary-thyroid axis. Compr. Physiol..

[4] Chen X., Zhou Y., Zhou M., Yin Q., Wang S. (2018). Diagnostic values of free triiodothyronine and free thyroxine and the ratio of free triiodothyronine to free thyroxine in thyrotoxicosis. Int. J. Endocrinol..

[5] Mortoglou A., Candiloros H. (2004). The serum triiodothyronine to thyroxine (T3/T4) ratio in various thyroid disorders and after Levothyroxine replacement therapy. Hormones.

[6] Chopra I.J., Smith S.R. (1975). Circulating thyroid hormones and thyrotropin in adult patients with protein-calorie malnutrition. J. Clin. Endocrinol. Metab..

[7] Favilla A.B., Horning M., Costa D.P. (2022). Advances in thermal physiology of diving marine mammals: The dual role of peripheral perfusion. Temperature.

[8] Williams T.M. (2022). Racing time: Physiological rates and metabolic scaling in marine mammals. Integr. Comp. Biol..

[9] Harrison R. (1969). Endocrine organs: Hypophysis, thyroid and adrenal. The Biology of Marine Mammals.

[10] Ridgway S.H., Patton G. (1971). Dolphin thyroid: Some anatomical and physiological findings. Z. Vgl. Physiol..

[11] Fair P.A., Montie E., Balthis L., Reif J.S., Bossart G.D. (2011). Influences of biological variables and geographic location on circulating concentrations of thyroid hormones in wild bottlenose dolphins (*Tursiops truncatus*). Gen. Comp. Endocrinol..

[12] Cowan D., Tajima Y. (2006). The thyroid gland in bottlenose dolphins (*Tursiops truncatus*) from the Texas Coast of the Gulf of Mexico: Normal structure and pathological changes. J. Comp. Pathol..

[13] Cowan D.F. (1966). Observations on the pilot whale Globicephala melaena: Organweight and growth. Anat. Rec..

[14] Mikaelian I., Labelle P., Kopal M., De Guise S., Martineau D. (2003). Adenomatous hyperplasia of the thyroid gland in beluga whales (*Delphinapterus leucas*) from the St. Lawrence Estuary and Hudson Bay, Quebec, Canada. Vet. Pathol..

[15] Garner M.M., Shwetz C., Ramer J.C., Rasmussen J.M., Petrini K., Cowan D.F., Raymond J.T., Bossart G.D., Levine G.A. (2002). Congenital diffuse hyperplastic goiter associated with perinatal mortality in 11 captive-born bottlenose dolphins (*Tursiops truncatus*). J. Zoo Wildl. Med..

[16] West K.L., Ramer J., Brown J.L., Sweeney J., Hanahoe E.M., Reidarson T., Proudfoot J., Bergfelt D.R. (2014). Thyroid hormone concentrations in relation to age, sex, pregnancy, and perinatal loss in bottlenose dolphins (*Tursiops truncatus*). Gen. Comp. Endocrinol..

[17] Nwachukwu C. (2020). Nutritional effects on fetal development during gestation in ruminants. Niger. J. Anim. Prod..

[18] Bazer F.W., Spencer T.E., Wu G., Cudd T.A., Meininger C.J. (2004). Maternal nutrition and fetal development. J. Nutr..

[19] Sullivan S.A. (2019). Hypothyroidism in pregnancy. Clin. Obstet. Gynecol..

[20] Leon A., Pillon C., Tebourski I., Bruyas J.F., Lupo C. (2023). Overview of the causes of abortion in horses, their follow-up and management. Reprod. Domest. Anim..

[21] Flower J.E., Allender M.C., Giovanelli R.P., Summers S.D., Spoon T.R., Leger J.A.S., Goertz C.E., Dunn J.L., Romano T.A., Hobbs R.C. (2015). Circulating concentrations of thyroid hormone in beluga whales (*Delphinapterus leucas*): Influence of age, sex, and season. J. Zoo Wildl. Med..

[22] St. Aubin D., Geraci J. (1989). Seasonal variation in thyroid morphology and secretion in the white whale, *Delphinapterus leucas*. Can. J. Zool..

[23] St. Aubin D., Ridgway S.H., Wells R., Rhinehart H. (1996). Dolphin thyroid and adrenal hormones: Circulating levels in wild and semidomesticated *Tursiops truncatus*, and influence of sex, age, and season. Mar. Mammal Sci..

[24] Suzuki M., Banno K., Usui T., Funasaka N., Segawa T., Kirihata T., Kamisako H., Ueda K., Munakata A. (2018). Seasonal changes in plasma levels of thyroid hormones and the effects of the hormones on cellular ATP content in common bottlenose dolphin. Gen. Comp. Endocrinol..

[25] Robeck T., Amaral R., da Silva V., Martin A., Montano G., Brown J. (2019). Thyroid hormone concentrations associated with age, sex, reproductive status and apparent reproductive failure in the Amazon river dolphin (*Inia geoffrensis*). Conserv. Physiol..

[26] Robeck T.R., Willis K., Scarpuzzi M.R., O’Brien J.K. (2015). Comparisons of life-history parameters between free-ranging and captive killer whale (*Orcinus orca*) populations for application toward species management. J. Mammal..

[27] Gislefoss R.E., Grimsrud T.K., Mørkrid L. (2015). Stability of selected serum hormones and lipids after long-term storage in the Janus Serum Bank. Clin. Biochem..

[28] Männistö T., Suvanto E., Surcel H.-M., Ruokonen A. (2010). Thyroid hormones are stable even during prolonged frozen storage. Clin. Chem. Lab. Med..

[29] Tjernvoll E., Åsberg A., Lian I.A., Løfblad L., Hov G.G., Thorstensen K., Mikkelsen G. (2025). Stability of 17 endocrine analytes in serum or plasma after four cycles of repeated freezing and thawing. Scand. J. Clin. Lab. Investig..

[30] Robeck T., Monfort S. (2006). Characterization of male killer whale (*Orcinus orca*) sexual maturation and reproductive seasonality. Theriogenology.

[31] Robeck T.R., Steinman K.J., O’Brien J.K. (2016). Characterization and longitudinal monitoring of serum progestagens and estrogens during normal pregnancy in the killer whale (*Orcinus orca*). Gen. Comp. Endocrinol..

[32] Robeck T.R., Blum J.L., Steinman K.J., Ratner J.R., Bergfelt D.R., O’Brien J.K. (2018). Longitudinal profiles of relaxin and progestagens during pregnancy, pregnancy loss and false pregnancy in the killer whale (*Orcinus orca*). Gen. Comp. Endocrinol..

[33] West B.T., Welch K.B., Galecki A.T. (2022). Linear Mixed Models: A Practical Guide Using Statistical Software.

[34] Zhang J., Yang Y., Ding J. (2023). Information criteria for model selection. Wiley Interdiscip. Rev. Comput. Stat..

[35] Field C.A., Welsh A.H. (2007). Bootstrapping clustered data. J. R. Stat. Soc. Ser. B Stat. Methodol..

[36] Horowitz G.L. (2008). Defining, Establishing, and Verifying Reference Intervals in the Clinical Laboratory: Proposed Guideline.

[37] Hennemann III W.W. (1983). Relationship among body mass, metabolic rate and the intrinsic rate of natural increase in mammals. Oecologia.

[38] White C.R., Marshall D.J., Alton L.A., Arnold P.A., Beaman J.E., Bywater C.L., Condon C., Crispin T.S., Janetzki A., Pirtle E. (2019). The origin and maintenance of metabolic allometry in animals. Nat. Ecol. Evol..

[39] Ashe E., Wray J., Picard C.R., Williams R. (2013). Abundance and survival of Pacific humpback whales in a proposed critical habitat area. PLoS ONE.

[40] Sung J., Kim J.-H. (2024). Association between ambient temperature and thyroid-stimulating hormone and free thyroxine levels in Korean euthyroid adults. Environ. Res..

[41] Burger A.G. (2004). Environment and thyroid function. J. Clin. Endocrinol. Metab..

[42] Eckhoff K.M., Maage A. (1997). Iodine content in fish and other food products from East Africa analyzed by ICP-MS. J. Food Compos. Anal..

[43] Haldimann M., Alt A., Blanc A., Blondeau K. (2005). Iodine content of food groups. J. Food Compos. Anal..

[44] Pinto C.A., de Castro Morais D., Franceschini S.d.C.C., Vieira Ribeiro S.A., Filomeno Fontes E.A., Pelucio Pizato N.M., Rocha de Faria F., Pereira R.J., Goés da Silva D., Abreu de Carvalho C. (2022). Iodine concentration in drinking water in the same or different seasons of the year in Brazilian Macroregions. J. Nutr. Metab..

[45] de Azevedo S.M., do Nascimento L.S., de Oliveira Silva L., de Almeida M.G., Azevedo L.S., Constantino W.D., Bastos W.R., Pestana I.A. (2023). Flood pulse as a driving force of Pb variation in four fish guilds from Puruzinho Lake (western Amazon). Environ. Sci. Pollut. Res..

[46] Delgiudice G.D., Mech L.D., Seal U.S. (1990). Effects of winter undernutrition on body composition and physiological profiles of white-tailed deer. J. Wildl. Manag..

[47] Zimmermann M.B., Boelaert K. (2015). Iodine deficiency and thyroid disorders. Lancet Diabetes Endocrinol..

[48] Schlumberger H.G. (1955). Spontaneous goiter and cancer of the thyroid in animals. Ohio J. Sci..

[49] Cabello G., Vilaxa A., Spotorno A.E., Valladares J., Pickard M., Sinha A., McArthur J., Behncke I., Duerr A., Sullivan R. (2003). Evolutionary adaptation of a mammalian species to an environment severely depleted of iodide. Pflügers Arch..

[50] Little G.J. (1991). Thyroid morphology and function and its role in thermoregulation in the newborn southern elephant seal (*Mirounga leonina*) at Macquarie Island. J. Anat..

[51] Walsh J.P. (2022). Thyroid function across the lifespan: Do age-related changes matter?. Endocrinol. Metab..

[52] Behringer V., Deimel C., Hohmann G., Negrey J., Schaebs F.S., Deschner T. (2018). Applications for non-invasive thyroid hormone measurements in mammalian ecology, growth, and maintenance. Horm. Behav..

[53] Ayres K.L., Booth R.K., Hempelmann J.A., Koski K.L., Emmons C.K., Baird R.W., Balcomb-Bartok K., Hanson M.B., Ford M.J., Wasser S.K. (2012). Distinguishing the impacts of inadequate prey and vessel traffic on an endangered killer whale (*Orcinus orca*) population. PLoS ONE.

[54] Todini L., Malfatti A., Valbonesi A., Trabalza-Marinucci M., Debenedetti A. (2007). Plasma total T3 and T4 concentrations in goats at different physiological stages, as affected by the energy intake. Small Rumin. Res..

[55] Fazio E., Bionda A., Chiofalo V., Crepaldi P., Lopreiato V., Medica P., Liotta L. (2022). Adaptive responses of thyroid hormones, insulin, and glucose during pregnancy and lactation in dairy cows. Animals.

[56] Almomin A.M.S., Mansour A.A., Sharief M. (2016). Trimester-specific reference intervals of thyroid function testing in pregnant women from Basrah, Iraq using electrochemiluminescent immunoassay. Diseases.

[57] Verstegen J., Dhaliwal G., Verstegen-Onclin K. (2008). Canine and feline pregnancy loss due to viral and non-infectious causes: A review. Theriogenology.

[58] Wu G., Bazer F., Wallace J., Spencer T. (2006). Board-invited review: Intrauterine growth retardation: Implications for the animal sciences. J. Anim. Sci..

[59] Robeck T.R., Gili C., Doescher B.M., Sweeney J., De Laender P., Van Elk C.E., O’Brien J.K. (2012). Altrenogest and progesterone therapy during pregnancy in bottlenose dolphins (*Tursiops truncatus*) with progesterone insufficiency. J. Zoo Wildl. Med..

[60] Parikh S.S., Kumar R., Patbandha T.K., Kumar P. (2024). Stillbirth. Periparturient Diseases of Cattle.

[61] Christianson W.T. (1992). Stillbirths, mummies, abortions, and early embryonic death. Vet. Clin. N. Am. Food Anim. Pract..

[62] Corriveau S., Blouin S., Raiche É., Nolin M.-A., Rousseau É., Pasquier J.-C. (2015). Levothyroxine treatment generates an abnormal uterine contractility patterns in an in vitro animal model. J. Clin. Transl. Endocrinol..

[63] Wei H., Guan Q., Yu Q., Chen T., Wang X., Xia Y. (2022). Assessing maternal thyroid function and its relationship to duration of the first stage of labor. Eur. Thyroid J..

[64] Bagheripuor F., Ghanbari M., Piryaei A., Ghasemi A. (2018). Effects of fetal hypothyroidism on uterine smooth muscle contraction and structure of offspring rats. Exp. Physiol..

[65] Cantón G.J., Navarro M.A., Asin J., Chu P., Henderson E.E., Mete A., Uzal F.A. (2023). Equine abortion and stillbirth in California: A review of 1,774 cases received at a diagnostic laboratory, 1990–2022. J. Vet. Diagn. Investig..

[66] Hecker Y.P., Gonzalez-Ortega S., Cano S., Ortega-Mora L.M., Horcajo P. (2023). Bovine infectious abortion: A systematic review and meta-analysis. Front. Vet. Sci..

[67] Shi Q., Wu M., Chen P., Wei B., Tan H., Huang P., Chang S. (2022). Criminal of adverse pregnant outcomes: A perspective from thyroid hormone disturbance caused by SARS-CoV-2. Front. Cell. Infect. Microbiol..

[68] Wajner S.M., Maia A.L. (2012). New insights toward the acute non-thyroidal illness syndrome. Front. Endocrinol..

[69] Pasternak J.A., MacPhee D.J., Harding J.C. (2020). Maternal and fetal thyroid dysfunction following porcine reproductive and respiratory syndrome virus2 infection. Vet. Res..

[70] Alberts M.K., McCann J.P., Woods P.R. (2000). Hemithyroidectomy in a horse with confirmed hyperthyroidism. J. Am. Vet. Med. Assoc..

[71] Bezzola P. (2002). Thyroid carcinoma and hyperthyroidism in a dog. Can. Vet. J..

[72] Petca A., Dimcea D.A.-M., Dumitrașcu M.C., Șandru F., Mehedințu C., Petca R.-C. (2023). Management of hyperthyroidism during pregnancy: A systematic literature review. J. Clin. Med..

[73] Petričević N., Shek Vugrovečki A., Milinković Tur S., Fruk S., Jurkić Krsteska G., Vince S., Belegu K., Muca G., Pejakivić Hlede J., Đuričić D. (2025). Thyroid hormones in female and male reproduction with special reference to dogs and cats. Vet. Stanica.

[74] De Groot L.J., Bartalena L., Feingold K.R. (2025). Thyroid storm. Endotext [Internet].

[75] Zimmerman C.F., Ilstad-Minnihan A.B., Bruggeman B.S., Bruggeman B.J., Dayton K.J., Joseph N., Moas D.I., Rohrs H.J. (2022). Thyroid storm caused by hyperemesis gravidarum. AACE Clin. Case Rep..

[76] Caron P. (2022). Management of thyrotoxicosis and pregnancy: Review of the current literature and an update of the care pathway. Proceedings of the Annales d’Endocrinologie.

[77] Krassas G., Karras S.N., Pontikides N. (2015). Thyroid diseases during pregnancy: A number of important issues. Hormones.

[78] Peterson M. (2012). Hyperthyroidism in cats: What’s causing this epidemic of thyroid disease and can we prevent it?. J. Feline Med. Surg..

[79] Leav I., Schiller A., Rijnberk A., Legg M., Der Kinderen P. (1976). Adenomas and carcinomas of the canine and feline thyroid. Am. J. Pathol..

[80] Bergfelt D.R., Thompson D.L., Brown J.L., Presley N.A., West K.L., Campbell M., Adams G.P. (2012). Investigation of an Immunoreactive Chorionic Gonadotropin-Like Substance in the Placenta, Serum, and Urine of Pregnant Bottlenose Dolphins (*Tursiops truncatus*). Aquat. Mamm..

[81] Pirahanchi Y., Toro F., Jialal I. (2018). Physiology, Thyroid Stimulating Hormone.

[82] Andersen S., Andersen S.L. (2024). Response to Fitzgerald et al. re:“Thyroid Stimulating Hormone and Thyroid Hormones (Triiodothyronine and Thyroxine): An American Thyroid Association-Commissioned Review of Current Clinical and Laboratory Status”. Thyroid.

