# Supplementary material for: Variations in Circulating Thyroid Hormone Profiles Across Age, Sex, and Pregnancy Outcomes in Killer Whales (Orcinus orca) Under Human Care
Source: Animals (Basel). 2026 Mar 13;16(6):907. doi: 10.3390/ani16060907 (PMC13023256; doi:10.3390/ani16060907)
Supplement: Supplementary file 1 [file animals-16-00907-s001.zip › animals-4140528-supplementary.pdf]

Supplemental Information (SI) for:

Variations in Circulating Thyroid Hormone Profiles Across Age, Sex, and Pregnancy Outcomes in Killer Whales (*Orcinus orca*) Under Human Care

Todd R. Robeck [1,2,*], Karen J. Steinman [1], Gisele A. Montano [1,2], Steve Paris [3], Janine L. Brown [3]

[1] Species Preservation Laboratory, SeaWorld Parks & Entertainment, Inc., San Diego, CA, United States
[2] United Parks and Resorts, Corporate Zoological Operations, Sea World Orlando, 32821
[3] Center for Species Survival, Smithsonian National Zoo & Conservation Biology Institute, 1500 Remount Road, Front Royal, VA 22630, USA

* Correspondence: Todd.Robeck@unitedparks.com; Tel.: +1-619-813-6828

This PDF file includes
Tables S1 to S5

Table S1. Assay validation statistics for thyroid hormone assays: Total triiodothyronine (T3); thyroxine (T4); and thyroid stimulating hormone (TSH). Assay validations included parallelism and accuracy check for two pools of killer whale serum samples, one female and one male. TSH parallelism did not pass validation threshold, so the accuracy/recovery was not performed.

	Hormone		
**Assay Validation**	**Total T3**	**Total T4**	**TSH**
Parallelism			
Linear regression	♀: y = 1.07x + 11.6<br>♂: y = 1.04x - 18	♀: y = 0.88x + 12<br>♂: y = 1.13x – 2.4	♀: y = 0.14x + 0.78<br>♂: y = 0.03x + 1.4
r^2	♀: 0.963<br>♂: 0.982	♀: 0.94<br>♂: 0.99	♀: 0.65<br>♂: 0.02
F statistic	♀: $F_{1,1}$ = 25.8<br>♂: $F_{1,1}$ = 54.8	♀: $F_{1,2}$ = 33.7<br>♂: $F_{1,1}$ = 1425.72	♀: $F_{1,3}$ = 5.6<br>♂: $F_{1,3}$ = 0.08
P-value	♀: 0.124<br>♂: 0.086	♀: 0.03<br>♂: < 0.02	♀: 0.1<br>♂: 0.8
Accuracy/Recovery			
Linear regression	♀: y = 0.99x – 36.5<br>♂: y = 1.15x – 77.6	♀: y = 0.96x – 1.5<br>♂: y = 1.03x – 1.5	Not performed
r^2	♀: 0.999<br>♂: 0.989	♀: 0.963<br>♂: 0.84	
F statistic	♀: $F_{1,1}$ = 9295.5<br>♂: $F_{1,1}$ = 88.9	♀: $F_{1,1}$ = 25.9<br>♂: $F_{1,1}$ = 5.2	
P-value	♀: < 0.02<br>♂: < 0.07	♀: 0.123<br>♂: 0.26	

Table S2. Evaluation of the random effects portion of the restricted maximum likelihood linear mixed model (LMM) analysis of the killer whale thyroid hormones total triiodothyronine (tT3), total thyroxine (tT4) and their ratio (tT3:tT4). The random effects was first evaluated to determine if model was improved using a 2 (animal id) or 3 (animal nested within location) level mixed effect model (REML). All fixed variables were included during the selection of the random portion of the models. Models containing variables for random slopes were then compared to models without only random intercepts. Significance of full and reduced models were assessed using LR test. Final models are in bold.

Hormone	Random intercept	Random slopes	Mixed vs linear model	LR test full vs reduced model	Comment
**tT3**	**Location[1], ID[2]**	**none**	**χ^2 = 540, P < 0.0001**		**Level 3 REML**
tT3	ID	none	χ^2 = 509, P = 0.0001	χ^2 = 31.0, P < 0.0001	Level 2 REML
tT3	Location, ID	ID, ID	χ^2 = 541, P < 0.0001	χ^2 = 1.3, P = 0.7279	No improvement
**tT3**	**Location, ID**	**group, group**	**χ^2 = 556, P < 0.0001**	**χ^2 = 16.3, P < 0.0001**	**Final level 3 model**
tT3	Location, ID	----, group	χ^2 = 556, P < 0.0001	χ^2 = 0.00, P = 1.0	No improvement
**tT4**	**Location, ID**	**none**	**χ^2 = 522 P <0.0001**		**Level 3 REML**
tT4	ID	none	χ^2 = 496, P < 0.0001	χ^2 = 26.6, P < 0.0001	Level 2 REML
tT4	Location, ID	ID, ID	χ^2 = 532, P < 0.0001	χ^2 = 10.3 P = 0.01	New full model
**tT4**	**Location, ID**	**group, group**	**χ^2 = 551, P < 0.0001**	**χ^2 = 29.8, P < 0.0001**	**Final Level 3 model**
tT4	Location, ID	ID, group	χ^2 = 551, P < 0.0001	χ^2 = 0.0, P = 1.0	No improvement
tT4	Location, ID	group, ID	χ^2 = 532, P < 0.0001	χ^2 = 19.5, P = <0.0000	No improvement
tT4	Location, ID	----, group	χ^2 = 551, P < 0.0001	χ^2 = 0.0, P = 1.0	No improvement
tT4	Location, ID	group, ----	χ^2 = 522, P < 0.0001	χ^2 = 30, P = <0.0000	No improvement
**tT3:tT4 ratio**	**Location, ID**	**none**	**χ^2 = 465 P <0.0001**		**Level 3 REML**
tT3:tT4 ratio	ID	none	χ^2 = 450, P < 0.0001	χ^2 = 15.0, P = 0.0001	Level 2 REML
tT3:tT4 ratio	Location, ID	ID, ID	χ^2 = 465, P < 0.0001	χ^2 = 0.22, P = 1.0	No improvement
tT3:tT4 ratio	Location, ID	----, ID	χ^2 = 465, P < 0.0001	χ^2 = 0.22, P = 0.90	No improvement
tT3:tT4 ratio	Location, ID	----, group	χ^2 = 498, P < 0.0001	χ^2 = 33.6, P < 0.0001	Improved > Level 3
**tT3:tT4 ratio**	**Location, ID**	**group, group**	**χ^2 = 498, P < 0.0001**	**χ^2 = 0.0, P = 1.0**	**Final Level 3 model**

[1]Location was a fixed variable labeled "park" and coded 0,1,2,3 for analysis and represented animals living at 1 of following 4 SeaWorld (SW) parks, California (SWC), Florida (SWF), Ohio (SWO), and Texas (SWT). Fixed variable "group" (coded as 0,1,2,3,4) represented: Juvenile (female < 8, Male < 13), early pregnancy (Day 2 through 178 post-conception [pc]), mid (Day 179 to day 356 pc) and late (day 357 until parturition, Robeck et al., 2018).

Table S3. Fixed variable selection for the 3-level restricted maximum likelihood linear mixed model (REML) analysis of the killer whale thyroid hormones total triiodothyronine (tT3), total thyroxine (tT4) and their ratio (tT3:tT4) with animal location (park) and animals as the random intercept variable, group as random slopes and residuals independent by group. Final fixed portion of the model was selection based on the model having the lowest AIC, BIC and CAIC. Fixed portion of model variables listed prior to parallel lines, random portions listed after.

Models	Model Wald χ^2	AIC	BIC	CAIC
**1) tT3 ~ c.air i.sex i.season i.group ||park:group, ||id:group,**	**χ^2 = 42, P < 0.0001**	**12768**	**12873**	**12893**
2) tT3 ~ i.sex i.season i.group ||park:group, ||id:group,	χ^2 = 40, P < 0.0001	13006	13105	13124
3) tT3 ~ i.season i.group ||park: group, ||id:group,	χ^2 = 39, P < 0.0001	13010	13104	13122
4) tT3 ~ i.group ||park: group, ||id:group,	χ^2 = 37, P < 0.0001	13012	13085	13099
**1) tT4 ~ c.air i.sex i.season i.group ||park:group, ||id:group,**	**χ^2 = 128, P < 0.0001**	**5004**	**5108**	**5128**
2) tT4 ~ i.sex i.season i.group ||park:group, ||id:group,	χ^2 = 141, P < 0.0001	5106	5201	5219
3) tT4 ~ i.season i.group ||park:group, ||id:group,	χ^2 = 142, P < 0.0001	5103	5188	5204
4) tT4 ~ i.group ||park:group, ||id:group,	χ^2 = 82, P < 0.0001	5146	5215	5227
**1) tT3:tT4 ~ c.air i.sex i.season i.group ||park:group, ||id:group,**	**χ^2 = 68, P < 0.0001**	**7279**	**7384**	**7404**
2) tT3:tT4 ~ i.sex i.season i.group ||park:group, ||id:group,	χ^2 = 67, P < 0.0001	7404	7499	7517
3) tT3:tT4 ~ i.season i.group ||park:group, ||id:group,	χ^2 = 64, P < 0.0001	7406	7496	7516
4) tT3:tT4 ~ i.group ||park:group, ||id:group,	χ^2 = 32, P < 0.0001	7428	7501	7515

AIC: Akaike's Information criterion, BIC: Bayesian information criterion, CAIC: consistent Akaike's information criterion

Air: monthly mean air temperature at the month of sample collection. group: Juvenile (female < 8, Male < 13), early pregnancy (Day 2 through 178 post-conception [pc]), mid (Day 179 to day 356 pc) and late (day 357 until parturition, Robeck et al., 2018).

Table S4. Evaluation of the random effects portion of the restricted maximum likelihood linear mixed model (LMM) analysis of the killer whale thyroid hormones, total triiodothyronine (tT3), total thyroxine (tT4) and their ratio (tT3:tT4) within normal and abnormal pregnancies. The random effects was first evaluated to determine if model was improved using a 2 (animal id) or 3 (animal nested within location) level mixed effect model (REML). All fixed variables were included during the selection of the random portion of the models. Models containing variables for random slopes were then compared to models without only random intercepts. Significance of full and reduced models were assessed using LR test. Final models are in bold.

Hormone	Random intercept	Random slopes	Mixed vs linear model	LR test full vs reduced model	Comment
**tT3**	**Location[1], ID[2]**	**none**	**χ^2 = 101, P < 0.0001**		**Level 3 REML**
tT3	ID	none	χ^2 = 79, P = 0.0001	χ^2 = 22.4, P < 0.0001	Level 2 REML
tT3	Location, ID	ID, ID	χ^2 = 102, P < 0.0001	χ^2 = -1.3, P = 1.0	No improvement
tT3	Location, ID	ID, pstage[3]	χ^2 = 114 P < 0.0001	χ^2 = 12.7, P = 0.0016	Improved over RI only
**tT3**	**Location, ID**	**----, pstage**	**χ^2 = 114, P < 0.0001**	**χ^2 = 0.0, P = 1.0**	**Final Random RI RS**
**tT4**	**Location, ID**	**none**	**χ^2 = 124, P <0.0001**		**Level 3 REML**
tT4	ID	none	χ^2 = 101, P < 0.0001	χ^2 = 22, P < 0.0001	Level 2 REML
tT4	Location, ID	ID, ID	χ^2 = 124, P < 0.0001	χ^2 = 0, P = 1.0	No improvement
tT4	Location, ID	pstage, pstage	χ^2 = 127, P < 0.0001	χ^2 = 3.44, P = 0.179	No improvement
tT4	Location, ID	----, pstage	χ^2 = 126, P < 0.0001	χ^2 = 3.44, P = 1.0	No improvement
tT3:tT4 ratio	Location, ID	none	χ^2 = 87, P <0.0001		Level 3 REML
**tT3:tT4 ratio**	**ID**	**none**	**χ^2 = 86, P < 0.0001**	**χ^2 = 0.45, P = 0.5016**	**Level 2 REML**
tT3:tT4 ratio	Location, ID	ID	χ^2 = 72, P < 0.0001	χ^2 = 0.22, P = 0.8939	No improvement
tT3:tT4 ratio	Location, ID	Pstage	χ^2 = 72, P < 0.0001	χ^2 = 0.08, P = 1.0	No improvement

[1]Location is a fixed variable labeled "park" and coded 0,1,2,3 for analysis and represented animal living at 1 of following 4 SeaWorld (SW) parks, California (SWC), Florida (SWF), Ohio (SWO), and Texas (SWT). [2]ID represented a unique animal identification number. RI: random intercept, RS: random slopes. [3]Fixed variable "pstage" (coded as 0,1,2) represents trimester of pregnancy: early pregnancy (Day 2 through 178 post-conception [pc]), mid (Day 179 to day 356 pc) and late (day 357 until parturition, Robeck et al., 2018). [2]ID represented a unique animal identification number. RI: random intercept, RS: random slopes.

Table S5. Fixed variable selection for the 3-level restricted maximum likelihood linear mixed model (REML) analysis of the killer whale thyroid hormones total triiodothyronine (tT3), total thyroxine (tT4) and their ratio (tT3:tT4) within pregnancy. Animal location (park) and animals as the random intercept variable and stage as random slopes. Final fixed portion of the model was selection based on the model having the lowest AIC, BIC and CAIC. Fixed portion of model variables listed prior to parallel lines, random portions listed after.

Models	Model Wald χ^2	AIC	BIC	CAIC
**1) tT3 ~ c.age i.season i.pstage i.pgresult i.pstage*i.pgresult ||park:, ||id:stage,**	**χ^2 = 48, P = 0.0005**	**3885**	**3887**	**3992**
2) tT3 ~ c.age i.season i.pstage i.pgresult ||park:, ||id: stage,	χ^2 = 37, P = 0.0002	3927	3984	3998
3) tT3 ~ c.age i.pstage i.pgresult ||park:, ||id:stage,	χ^2 = 30, P < 0.0001	3939	3940	3995
4) tT3 ~ i.pstage i.pgresult ||park:, ||id:stage,	χ^2 = 14, P = 0.1	3951	3952	4002
**1) tT4 ~ c.age i.season i.pstage i.pgresult i.pstage*i.pgresult ||park:, ||id:,**	**χ^2 = 126, P < 0.0001**	**1477**	1554	1573
2) tT4 ~ c.age i.season i.pstage i.pgresult ||park:, ||id:,	χ^2 = 88.8, P < 0.0001	1498	1550	1563
3) tT4 ~ c.age i.pstage i.pgresult ||park:, ||id:,	χ^2 = 64, P < 0.0001	1508	1548	1558
4) tT4 ~ i.pstage i.pgresult ||park:, ||id:,	χ^2 = 55, P < 0.0001	1508	**1544**	**1553**
1) tT3T4 ~ c.age i.season i.pstage i.pgresult i.pstage*i.pgresult ||id:,	χ^2 = 66, P < 0.0001	-1372	-1298	-1280
**2) tT3T4 ~ c.age i.season i.pstage i.pgresult ||id:,**	**χ^2 = 60, P < 0.0001**	**-1417**	-1367	-1355
**3**) tT3T4 ~ i.season i.pstage i.pgresult ||id:,	χ^2 = 21, P < 0.0001	-1412	-1375	-1367
4) tT3T4 ~ i.pgstage i.pgresult ||id:,	χ^2 = 18, P < 0.0001	-1426	**-1393**	**-1386**

AIC: Akaike's Information criterion, BIC: Bayesian information criterion, CAIC: consistent Akaike's information criterion.

[3]Fixed variable "pstage" (coded as 0,1,2) represents trimester of pregnancy: early pregnancy (Day 2 through 178 post-conception [pc]), mid (Day 179 to day 356 pc) and late (day 357 until parturition, Robeck et al., 2018). Pgresult: (coded 0, 1, 2, 3, 4) represents normal pregnancy, production of a live calf that survived beyond 30 days; failure to thrive, live calves that died before 30 days; stillbirth, full-term calves born dead or that died within 24 hours; dystocia, stillborn calf within normal gestation length but clinically difficult labor; and aborted calf, dead calf born before the minimum gestation length (485 days). [27].
